# Role of the Transcriptional Repressor BCL6 in Allergic Response and Inflammation

**DOI:** 10.1097/WOX.0b013e31817dc522

**Published:** 2008-07-15

**Authors:** Masafumi Arima, Takeshi Fukuda, Takeshi Tokuhisa

**Affiliations:** 1 Department of Developmental Genetics, Chiba University Graduate School of Medicine, Chiba; 2 Department of Pulmonary Medicine and Clinical Immunology, Dokkyo University School of Medicine, Dokkyo, Japan

**Keywords:** allergy, BCL6, cytokine, chemokine, lgE, Th2, transcriptional repressor

## Abstract

Various molecules participate in different phases of allergic reactions. This means that many genes are encoding molecules related to allergic reactions, such as cytokines, chemokines, and their receptors as effector molecules. The transcriptional repressor BCL6 has emerged as a multifunctional regulator of lymphocyte differentiation and immune responses. BCL6-deficient (BCL^-/-^) mice display T helper type 2 (Th2)-type inflammation, which is caused by abnormality of both lymphoid cells and nonlymphoid cells. Thus, BCL6 apparently contributes to negative regulation of various central molecules such as cytokines, in particular Th2 cytokines, CC chemokines, and immunoglobulin E in allergic diseases. Therefore, BCL6 may be a molecular target for Th2-type allergic diseases.

## 

Allergic diseases are characterized by elevated serum immunoglobulin E (IgE) levels and hypersensitivity to normally innocuous antigens (allergens). A particular direct allergen first encounters antigen-processing cells such as dendritic cells (DCs) or macrophages. The allergen is captured by the antigen-processing cells, processed, and presented to CD4^+ ^T cells. CD4^+ ^T cells are polarized into T helper type 1 (Th1) cells, producing interferon-γ and interleukin 2 (IL-2), and Th2 cells, producing IL-4, IL-5, IL-6, IL-10, and IL-13. Interleukin 4 and IL-13 stimulate immunoglobulin class switching, leading to the production of IgE, which binds to its high-affinity receptor (FcϵRI) on the surface of mast cells or basophils. The association of captured allergens with IgE bound to FcϵRI on the cell surface activates signal transduction in the cells and rapidly leads to the release of inflammatory cytokines and chemical mediators, such as histamine and leukotrienes. The Th2-type cytokines also trigger the production of chemokines in tissue fibroblasts or epithelial cells, promoting the infiltration of inflammatory cells into sites exposed to allergens.

Various allergy-related molecules are basically controlled by transcriptional regulators, critical molecules believed to govern the pathogenesis of allergic diseases by regulating cytokine production, mediator synthesis, and IgE production at gene expression levels. Recent genetic studies have demonstrated that transcriptional factors and regulators are involved in the development of allergic diseases. The molecular mechanisms of allergic diseases, in particular bronchial asthma, are gradually becoming clearer. However, nearly all previous studies of transcriptional factors have focused on active regulators, including GATA binding protein 3 (GATA3), signal transducer and activator of transcription 6 (STAT6), c-Maf, NF-AT, NF-κB, and c-fos. The functional roles of transcriptional repressors in allergic diseases remain poorly understood. Because transcriptional repressors may have an important role in tuning physiologically optimal transcription, functional failure of tightly controlled constitutive mechanisms regulated by a given repressor may lead to the development of allergic diseases.

A transcriptional repressor gene, *BCL6*, has emerged as a multifunctional regulator of lymphocyte differentiation and immune responses [1,2]. BCL6 mutant mice display two prominent phenotypes: failure to form germinal centers during T cell-dependent immune responses and fatal eosinophilic inflammatory diseases characterized by the presence of Th2 cells and mast cells [3-5]. Although the molecular mechanisms underlying these phenotypes are largely unknown, studies in BCL6-deficient mice have suggested that BCL6 functions to prevent the development or attenuate the pathogenesis of allergic diseases. In this article, we review the functions of BCL6 in allergic diseases by focusing on recent data from our laboratories and from other groups.

## Structure and Basic Functions of BCL6

**Figure 1:**
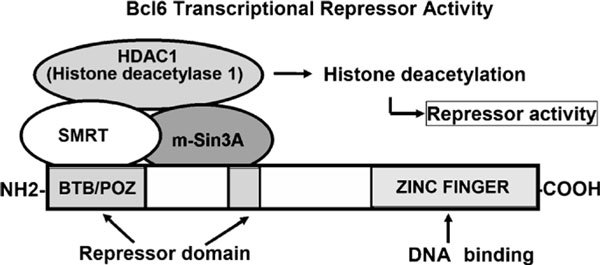
**Structure of BCL6**. The *BCL6 *gene encodes a 92- to 98-kd nuclear phosphoprotein that contains the BTB/POZ domain in the NH_2_-terminal region and Krüppel-type zinc finger motifs in the COOH-terminal region. The NH_2_-terminal half of BCL6 can bind to SMRT and recruit the SMRT/histone deacetylase complex to silencer regions of target genes to repress the expression of these genes. Because the BCL-6 zinc-fingers bind DNA in a sequence-specific manner, *BCL6 *can function as a sequence-specific transcriptional repressor.

**Figure 2:**
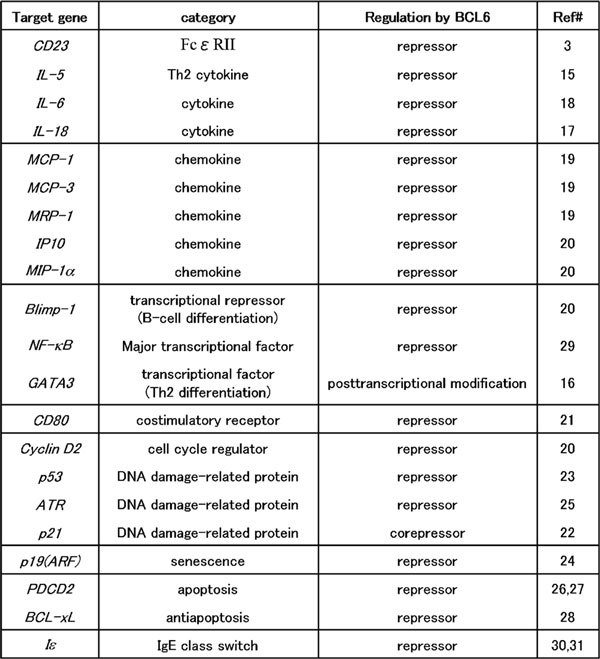
**Target genes of BCL6**. Previous studies indicate that BCL6 acts as a multipotential molecule because its target genes are for various molecules, including cytokines, chemokines, cell cycle regulators, DNA damage-related proteins, apoptosis-related proteins, and transcriptional factors. ATR indicates ataxia telangiectasia and Rad3 related; IP10, interferon-inducible protein 10; MIP-1α, macrophage inflammatory protein 1α; MRP-1, macrophage inflammatory protein-related protein 1; PDCD2, programmed cell death 2.

The human proto-oncogene *BCL6 *was first identified in studies of chromosomal breakpoints involving 3q27 in diffuse large B-cell lymphomas [6-8]. BCL6 is expressed at low levels in a wide variety of tissues. It is expressed abundantly only in germinal center B cells, cortical thymocytes, and parafollicular T cells within secondary lymphoid tissues [9]. High BCL6 levels are also present in cells of monocytic lineage [10]. The *BCL6 *gene encodes a 92- to 98-kd nuclear phosphoprotein that contains the BTB/POZ domain in the NH2-terminal region and Krüppel-type zinc finger motifs in the COOH-terminal region (Figure 1). Because the NH_2_-terminal half of BCL6 can bind to silencing mediator of retinoid and thyroid receptor protein (SMRT) and recruit the SMRT/histone deacetylase complex to silencer regions of target genes to repress the expression of these genes, *BCL6 *can function as a sequence-specific transcriptional repressor. The BCL6 zinc-fingers bind to DNA in a sequence-specific manner, and a consensus BCL6 DNA-binding site has been identified [11,12]. The BCL6 consensus binding site resembles the GAS motif recognized by the STAT family of transcription factors, raising speculation that BCL6 may bind competitively to some STAT-binding sites to repress expression of STAT-dependent genes [3,13,14]. Target genes of BCL6 have been identified. Available evidence indicates that BCL6 is a multipotential molecule because its target genes are related to various molecules, including cytokines, chemokines, cell cycle regulators, DNAdamage-related proteins, apoptosis-related proteins, and transcriptional factors (Figure 2) [3,15-31].

When overexpressed, BCL6 can inhibit cell growth and even induce programmed cell death in nonlymphoid cells such as fibroblasts [32,33]. This finding indicates that BCL6 is toxic to cells that do not normally express the protein at high levels. Although this fact suggests an important role of BCL6 in normal cell growth, its relevance to normal cell physiology is unclear. We have recently demonstrated that BCL6 is essential for the generation of high-affinity memory B cells in germinal centers[4,34] and that BCL6 controls the generation and maintenance of memory CD8^+ ^T cells[35,36] and memory CD4 T cells [37]. Our findings suggest that Bcl6 in T cells plays a role in protecting memory precursor T cells from apoptosis and may be involved in the survival of long-term memory T cells.

## Functions of BCL6 in B Cells

Although *BCL6 *messenger RNA (mRNA) can be detected in many tissues,[38] its protein expression is limited mainly to lymphocytes [38-40]. *BCL6 *expression is strongly induced in splenic B cells stimulated with IL-4 or IL-21 [41]. Although IL-2 and IL-7 mediate their biological effects through a common cytokine receptor gamma chain, which is shared with receptors for IL-4 and IL-21, stimulation with these cytokines did not induce high *BCL6 *expression in splenic B cells. Kinetic analysis demonstrated that *BCL6 *expression was transiently induced in B cells 30 minutes after stimulation with IL-4 or IL-21. *BCL6 *expression was further induced in these activated B cells within 6 hours after stimulation with IL-21. In contrast, the recurrence of IL-4Yinduced BCL6 expression was delayed, occurring 24 hours after stimulation. These findings suggested that IL-4 and IL-21 might be required for high *BCL6 *expression in B cells at different stages. Stimulation of splenic B cells with IL-4 or IL-21 induced high *BCL6 *expression with activation of STAT6 or STAT3, respectively, and binding of these STATs to the STAT/BCL6-binding sites in the BCL6 promoter was detected in germinal center B cells in mice. Interleukin 4 and IL-21 may thus have physiological roles in promoting high *BCL6 *expression in germinal center B cells, and BCL6 induced by STATs activated with IL-4 and/or IL-21 might autoregulate its promoter by negative feedback inhibition.

BCL6 can repress the IL-4Ydependent induction of immunoglobulin germ line transcripts by STAT6. Consistent with the role of BCL6 in modulating transcription from the germ line promoter, BCL6^-/- ^mice display an increased ability to class switch to IgE in response to IL-4 in vitro [30,31]. These animals also exhibit a multiorgan inflammatory disease characterized by the presence of large numbers of IgE^+ ^B cells. The apparent regulation of IgE production is abolished in STAT6^-/- ^BCL6^-/- ^mice, indicating that BCL6-dependent regulation of immunoglobulin class switching is STAT6 dependent. Thus, BCL6 can modulate the transcription of selective STAT6-dependent IL-4 responses, including IgE class switching in B cells. Furthermore, we recently simultaneously stimulated activated B cells with IL-4 and IL-21 and found that expression of Cγ1 germ line transcript (for IgG1 class switching) in BCL6 wild type (Wt) and BCL6^-/- ^B cells was enhanced by IL-21 stimulation, indicating that IL-21 is an enhancer of Cγ1 expression induced by IL-4 [42]. The amount of Cγ1 germ line transcript in the BCL6^-/- ^B cells was greater than that in the BCL6-Wt B cells. Conversely, IL-21 stimulation suppressed Cϵ (for IgE class switching) expression in the BCL6-Wt B cells. In contrast, such suppression was not observed in the BCL6^-/- ^B cells, suggesting that the IL-21Ymediated suppression of Cϵ. expression is caused by BCL6. Thus, BCL6 controls Cγ1 and Cϵ expression and stabilizes class switching to IgG1 in activated B cells simultaneously stimulated with IL-4 and IL-21.

CD23, also known as Fc epsilon RII or FcϵRII, is the low-affinity receptor for IgE. This antibody isotype is involved in allergy and resistance to parasites and has an important role in the regulation of IgE levels. Unlike many other antibody receptors, CD23 is a C-type lectin. It is found on not only mature B cells, but also activated macrophages, eosinophils, follicular DCs, and platelets. BCL6 also binds to the STAT6 DNA-binding sequence in the CD23b promoter to repress IL-4Yinduced activation of CD23 expression [3]. Therefore, BCL6 may be involved in the control of IgE production and act as a negative regulator on one of its ligands.

## Roles of BCL6 in T-Cell Function

BCL6^-/- ^mice show inflammatory responses in multiple organs, especially the heart and lung, characterized by eosinophil infiltration in young adults [3,5,41,43]. BCL6 is considered to regulate Th2-cell differentiation and/or Th2 cytokine production after differentiation. Several groups have investigated the roles of Th2-cell function.

Similar to the key regulators T-bet for Th1 and GATA3 for Th2, human BCL6 was differentially and reciprocally regulated during early Th1- and Th2-cell polarization. At the protein level, the expression of BCL6 was barely detectable in T-helper precursor cells. At the mRNA level, *BCL6 *is preferentially expressed in cells induced to polarize in the Th1 direction. BCL6 is expressed within 6 hours, and are maintained for at least 48 hours. In contrast, a low level of BCL6 expression is seen in cells induced to polarize in the Th2 direction, apparently because IL-4 down-regulates the human *BCL6 *gene at an early stage [44,45]. The differences in BCL6 between Th1- and Th2-polarization decreased or vanished 48 hours after each skewing.

Similar to human BCL6 during the early stage of differentiation into helper T cells, murine BCL6 protein is rapidly induced in naive T cells induced to polarize in the Th1 direction, whereas it is induced at a much lower level in T cells skewed toward the Th2 phenotype rather than the Th1 phenotype. The induction of early *BCL6 *gene expression was transient. Interestingly, Th2-skewed cells showed reinduction of BCL6 at a moderately higher level than Th1-skewed cells did within 7 days during the differentiation of each cell type [46]. We also found that BCL6 protein was transiently degraded in polarized Th2 cells by T-cell receptor-mediated restimulation, followed by another transient increase in BCL6 protein after 24 hours. In addition, *BCL6 *gene expression was inhibited by anti-IL-4 antibodies in polarized Th2 cells after restimulation. Thus, IL-4 may have a bipotential effect and attenuate as well as augment BCL6 production. The machinery for down-regulation of BCL6 remains unclear, whereas its up-regulation might involve STAT6-mediated activation of BCL6 promoter in B cells. Furthermore, autoregulation of BCL6 promoter by negative feedback inhibition may occur subsequently, as seen in B cells. Therefore, IL-4 may participate physiologically in the differentiation and reactivation of Th2 cells by tuning the expression of BCL6, which functions on target gene by competing with STATs.

Differentiation of the Th2 pathway has a critical role in determining the outcomes of immune responses. Mediators of Th2 differentiation have been partly elucidated and include a number of cytokines and related signaling molecules. Antigen triggering of naive T cells in the presence of IL-4 generates a Th2 response. Interleukin 4-induced gene activation in the Th2 response is mediated by STAT6 [47]. Dent et al[48] have shown that IL-4^-/-^BCL6^-/- ^and STAT6^-/-^BCL6^-/-^double-mutant mice have the same Th2-type inflammation of the heart and lungs as that characteristically occurring in BCL6^-/- ^mice. Furthermore, the Th2 cytokine response that develops in STAT6^-/-^BCL6^-/- ^and IL-4^-/-^BCL6 mice is similar to that in BCL6^-/- ^mice after immunization with a conventional antigen in adjuvant. In contrast to these in vivo findings, STAT6 was required for the in vitro differentiation of BCL6^-/- ^T cells into Th2 cells. BCL6 can bind to similar DNA-binding motifs as STAT transcription factors, suggesting that BCL6 regulates Th2 responses in vivo through a pathway(s) unrelated to IL-4 and STAT6. Kusam et al[16] have investigated the mechanism of BCL6 regulation during Th2-cell differentiation in vitro and have found that IL-6 signaling can promote dramatically increased levels of Th2 differentiation in BCL6^-/- ^CD4 T cells, as compared with BCL6-Wt CD4 T cells. Interleukin 6 could induce Th2 cytokine expression in STAT6^-/-^BCL6^-/- ^cells, but not in BCL6^-/- ^cells. They also studied whether GATA3 levels are up-regulated in STAT6^-/-^BCL6^-/- ^CD4 T cells as compared with STAT6^-/- ^CD4 T cells. Retrovirus-mediated expression of BCL6 in both STAT6^-/-^BCL6^-/- ^and BCL6-Wt T cells led to a significant decrease in GATA3 protein levels, thought to be regulated at a post-transcriptional level. Thus, overproduction of Th2 cytokines by BCL6^-/- ^T cells cannot be explained by loss of competitive inhibition of STAT6 activity, and the regulation of GATA3 protein levels by BCL6 is probably a key mechanism by which BCL6 regulates STAT6-independent Th2 cytokine expression and Th2 differentiation. The factors that control this alternative pathway have yet to be identified.

In vitro Th2 cells that have differentiated from BCL6-Wt and BCL6^-/- ^naive T cells produce cytokines other than IL-5 at similar levels after activation, respectively. Interleukin 5 is an important Th2 cytokine involved in controlling the growth, differentiation, and activation of eosinophils,[49] which densely infiltrate peripheral tissues in BCL6^-/- ^mice. Because production of IL-5 by BCL6^-/- ^T cells was augmented in vivo and in vitro, we have investigated whether BCL6-related mechanisms regulate IL-5 production, and have identified a BCL6-binding DNA sequence as a silencer (IL5BS) in the 3' untranslated (3'UT) region of the murine and human *IL-5 *genes [15].

As described above, regulation of IL-4 production by in vitro-differentiated Th2 cells is most likely not regulated by BCL6. However, we have identified putative multiple BCL6-binding sites in the IL-4 gene, and BCL6 binds to these sites at rest but not after activation (M. Arima, MD, PhD, et al, unpublished data, 2008). The physiological roles of BCL6 in lymphocytes with respect to Th2-cell differentiation and the functions of BCL6 such as cytokine production thus remain to be determined.

## Roles of BCL6 in Chemokine Production

Toney et al[19] have suggested that the development of Th2-type inflammation depends on BCL6 deficiency in some nonlymphoid tissues. They found that three chemokines, monocyte chemoattractant protein 1 (MCP-1), MCP-3, and macrophage inflammatory protein-related protein 1, were induced at higher levels in BCL6^-/- ^macrophages than in their BCL6-Wt counterparts. Furthermore, they identify three potential BCL6-binding sites in the promoter of each chemokine. Recently, we found that production of chemokines, including MCP-1, was regulated in airway epithelial cells and macrophages by BCL6. Many CC-type chemokine genes are located in a gene cluster at chromosomes 17q11.2 and 11q21.2 in humans and mice, respectively. The cluster includes genes encoding *CCL2 (MCP-1) -CCL7 (MCP-3) -CCL11 (eotaxin) -CCL8 (MCP-2) -CCL13 (MCP-4) -CCL1 (I-309) *in humans and *CCL2-CCL7-CCL11-CCL12 (MCP-5)-CCL8-CCL1 *in mice. The strong similarity in the clusters between humans and mice strongly suggests that chemokine expression is orchestrated by the conserved machinery. Because *MCP-1 (CCL2) *is known to be negatively regulated by BCL6 and some putative BCL6-binding sequences are observed in the gene clusters, we examined the role of BCL6 in the expression of these chemokine genes in the cluster. The levels of these chemokine mRNAs other than *CCL12 *mRNA were strikingly higher in lung tissues and bronchoalveolar lavage fluid cells from BCL6^-/- ^mice than in those from control mice. Down-regulation of BCL6 in human alveolar epithelial cells (A549) up-regulated the expression of these chemokine genes. Furthermore, chromatin modifications, such as acetylation and methylation of histone, and BCL6 binding to the sequences in the cluster were observed in each cell line on chromatin immunoprecipitation assay (T. Seto, MD, PhD, et al., unpublished data, 2008). These results suggest that BCL6 promotes Th2 differentiation via other mediators, such as chemokines, in addition to IL-4. This notion is supported by evidence that MCP-1 stimulates IL-4 production by T cells,[50] MCP-1^-/- ^mice show very poor Th2 differentiation,[51] and MCP-1 also suppresses IL-12 secretion from human peripheral blood monocytes [52]. Thus, BCL6 may play an important role in the coordinated regulation of these chemokine genes in pulmonary epithelial cells and macrophages. BCL6 apparently acts as a transcriptional repressor not only for Th2-cell functions, but also for migration of inflammatory cells, including eosinophils, macrophages, and mast cells. These cells also contribute to Th2-type inflammation and/or Th2-cell differentiation by producing Th2-type cytokines.

## Functions of BCL6 in Macrophages

BCL6 has been reported to play an important role in the functions of macrophages, as described previously. Furthermore, we have recently identified other effects of BCL6 on IL-18 production in macrophages. Interleukin 18 was discovered as a potent interferon-γ-inducing factor that is produced by macrophages and DCs in response to stimulation with microbes or microbe products [53]. Recent studies have shown that IL-18 can also promote Th2 cytokine production from T cells, natural killer cells, basophils, and mast cells [54,55]. Interleukin 18 seems to be involved in human allergic diseases such as atopic dermatitis (AD), bronchial asthma, and rhinitis. Several studies have reported correlations among serum levels of IL-18, IgE, and disease severity in AD and asthma [56-59]. Furthermore, leukocytes prepared from the peripheral blood of patients with AD produce larger amounts of IL-18 in response to lipopolysaccharide (LPS) than do leukocytes from healthy volunteers [60]. Interleukin 18 levels in nasal secretions are also higher in patients with allergic rhinitis than in healthy volunteers [61].

Because IL-18 triggers the differentiation of Th2 cells, we examined the expression of *IL-18 *mRNA in bone marrow-derived macrophages from BCL6^-/- ^mice after LPS stimulation [17]. The expression of IL-18 was strikingly up-regulated after stimulation. The expression was also up-regulated in RAW264 cells, a murine macrophage cell line, by transfection with the dominant negative type of *BCL6 *gene. We identified a putative BCL6-binding DNA sequence (IL-18BS) upstream of exon 1 of the murine *IL-18 *gene and three IL-18BSs in the promoter region of the human *IL-18 *gene. Binding of BCL6 in nuclear protein from resting RAW264 cells to murine IL-18BS was detected on gel retardation assay and chromatin immunoprecipitation assay. The binding activity decreased gradually in RAW264 cells after LPS stimulation. However, the amount of BCL6 protein in these cells remained constant over the period examined, suggesting the functional modification of BCL6 protein after stimulation. Furthermore, murine IL-18BSwas required forBCL6to repress the expression of the luciferase reporter gene under control of the IL-18 promoter. Therefore, BCL6 might be a key regulator of IL-18 production by macrophages in allergic diseases.

## Roles of BCL6 in Mast Cells

Mast cells are distributed throughout vascularized tissues and play a key role in allergic reactions by releasing proinflammatory mediators. Mast cell-derived factors act on immune-competent cells to promote survival of eosinophils, maturation of DCs, and activation of B and T cells by producing cytokines such as Th2 cytokines and chemokines such as MCP-1,[62-66] macrophage inflammatory protein 1α, regulated on activation normal T-cell expressed and secreted (RANTES), and eotaxin, which foster Th2 development and eosinophil recruitment [67]. Available evidence thus suggests that mast cells are an initiator of Th2-type inflammation in BCL6^-/- ^mice. We have therefore investigated the properties and functions of BCL6^-/- ^mast cells derived from bone marrow cells cultured with IL-3 (BMMCs). There was no significant difference between BCL6^-/- ^BMMCs and BCL6- Wt BMMCs in the expression of surface FcR1 or c-Kit, in apoptosis induced by deprivation of IL-3, or in degranulation. However, BCL6^-/- ^BMMCs stimulated by FcRI/IgE crosslinking expressed greater amounts of mRNAs of Th2 cytokines such as *IL-4, IL-5*, and *IL-13 *than BCL6-Wt BMMCs did. Because Th2 cytokines, including IL-4 and IL-13, have an important role in Th2 development,[68,69] mast cells may be one of the initiators of Th2 dominance in BCL6^-/- ^mice. Because the number of intraepithelial mast cells increased in BCL6^-/- ^mice[19] and the production of Th2 cytokines was augmented in activated BCL6^-/- ^mast cells, BCL6^-/- ^mast cells may also be a major effector of Th2-type inflammation in BCL6^-/- ^mice. Thus, BCL6^-/- ^mast cells are one of the initiators of Th2-type inflammation in BCL6^-/- ^mice.

## Roles of BCL6 in Asthma

Interestingly, some BCL6-regulated genes are representative genes implicated in allergic diseases. These genes are critical to the pathogenesis of allergic diseases such as asthma and pollinosis. Recently, we studied the roles of BCL6 in T cells in the development of asthma. To this end, Lck promoter-controlled BCL6 transgenic (Lck-BCL6-Tg) mice and BCL6-Wt mice were sensitized with intraperitoneal injections of ovalbumin. Fourteen days after the antigen challenge, isolated Th2 cells (T1ST2 positive) were stimulated with anti-CD3 antibody. Production of IL-4 by T1ST2 T cells from Lck-BCL6 Tg mice was significantly attenuated as compared with that by BCL6-Wt mice, indicating that Th2 cytokine production was inhibited by BCL6 in T cells (T. Ogasawara, MD, PhD, et al, unpublished data, 2008). We also investigated the effect of T cells overexpressing BCL6 on the development of asthma by transferring splenic CD4 T cells from sensitized mice into nontreated BCL6-Wt B6 mice. Twenty-four hours later, these mice were challenged with ovalbumin. Antigen-induced eosinophilic airway inflammation was observed after transfer of lymphocytes from sensitized BCL6-Wt mice, associated with an elevated IL-4 level in bronchoalveolar lavage fluid after antigen exposure. The airway response and increased IL-4 production were prevented by transfer of lymphocytes from sensitized Lck-BCL6 Tg mice (T. Ogasawara, et al, unpublished data, 2008). Therefore, BCL6 may prevent the development of asthma by attenuating Th2 cytokine production in an experimental murine model, and failure of BCL6 repressor activity may be involved in the initiation and/or exacerbation of asthma in humans.

To test the hypothesis that variants of the *BCL6 *gene might be associated with atopic diseases, Adra et al[70] conducted a large-scale association study in both British (n = 275) and Japanese (n = 400) subjects. To identify the exact localization of the known *Hin*dIII polymorphism in the first intron of the *BCL6 *gene (7 Kb), they first fully sequenced the 7 Kb clone and determined primer sequences flanking the *Hin*dIII polymorphism. They analyzed relations between different types of atopic phenotypes (distinguished by serum IgE levels) and *BCL6 *genotypes. Variants of the *BCL6 *gene were found to be significantly associated with marked atopy, characterized by high IgE levels and positive radioallergo-sorbent test scores (> 3) to both house-dust mite and grass pollen mixtures. However, the functions of the variant of BCL6 remain unclear.

## Conclusions

**Figure 3:**
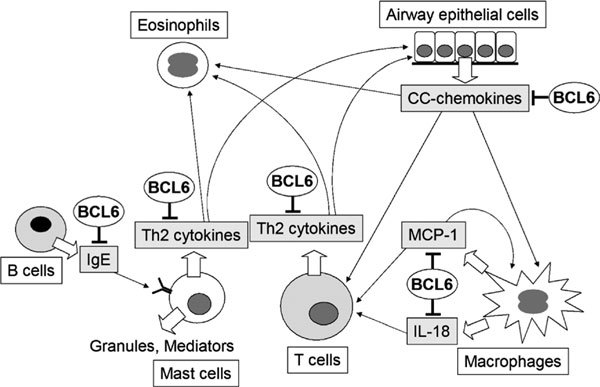
**Hypothetical role of BCL6 in allergic diseases**. BCL6 contributes to the function of a wide range of cells, including T cells, B cells, macrophages, mast cells, and airway epithelial cells to promote the pathogenesis of allergic diseases by regulating genes that control cytokine production, mediator synthesis, and IgE production.

In allergic diseases, IL-4-controlled transcriptional regulatory processes might be physiologically regulated by dual molecules, including activators such as STATs and repressors such as BCL6. This control mechanism can be likened to the concept of "yin and yang," involving two primal opposing, but complementary principles. This concept is plausible and critical for preserving biological homeostasis, in which BCL6 may participate in resetting genetic activity. For example, because Th2 cells secrete IL-4 and favor humoral immunity to extracellular pathogens,[71] BCL6 might attenuate the response. Given that a failure of immune homeostasis leads to the development of allergic diseases, BCL6 may play an important part in such diseases, acting as an inhibitor or protector. Thus, an imbalance between STAT6 and BCL6 may be critically involved in the development of allergic diseases because BCL6 contributes to the function of a wide range of cells, including T cells, B cells, macrophages, mast cells, and airway epithelial cells (Figure 3). In these cells, BCL6 contributes to the negative regulation of various key genes that predispose patients to allergies. Therefore, BCL6 may be a major molecular target for Th2-type allergic diseases. However, many important questions remain unresolved, despite considerable progress in understanding gene regulation by BCL6. Further genetic and functional analyses are needed to clarify whether *BCL6 *is related to the pathogenesis of allergic diseases, with the ultimate goal of establishing more effective means of prophylaxis and therapy. We hope that the studies presented in this review will provide new insights into allergic diseases.

## Competing interests

The authors declare that they have no competing interests.

## References

[B1] Dalla-Favera R Migliazza A Chang CC Niu H Pasqualucci L Butler M Molecular pathogenesis of B cell malignancy: the role of BCL-6 Curr Top Microbiol Immunol 1999 1 257 263 10.1007/978-3-642-60162-0_32 10396064

[B2] Staudt LM Dent AL Shaffer AL Yu X Regulation of lymphocyte cell fate decisions and lymphomagenesis by BCL-6 Int J Immunol 1999 1 381 403 10.3109/08830189909088490 10626250

[B3] Dent AL Shaffer AL Yu X Allman D Staudt LM Control of inflammation, cytokine expression, and germinal center formation by BCL-6 Science 1997 1 589 592 10.1126/science.276.5312.589 9110977

[B4] Fukuda T Yoshida T Okada S Hatano M Miki T Ishibashi K Disruption of the *Bcl6 *gene results in an impaired germinal center formation J Exp Med 1997 1 439 448 10.1084/jem.186.3.439 9236196 PMC2199007

[B5] Ye BH Cattoretti G Shen Q Zhang J Hawe N de Waard R The BCL-6 proto-oncogene controls germinal-centre formation and Th2-type inflammation Nat Genet 1997 1 161 170 10.1038/ng0697-161 9171827

[B6] Kerchaert JP Deweindt C Tilly H Quief S Lecocq G Bastard C LAZ3, a novel zinc-finger encoding gene, is disrupted by recurring chromosome 3q27 translocations in human lymphomas Nat Genet 1993 1 66 70 10.1038/ng0993-66 8220427

[B7] Ye BH Lista F Lo Coco F Knowles DM Offit K Chaganti RS Alterations of a zinc finger-encoding gene, *BCL-6*, in diffuse large-cell lymphoma Science 1993 1 747 750 10.1126/science.8235596 8235596

[B8] Miki T Kawamata N Hirosawa S Aoki N Gene involved in the 3q27 translocation associated with B-cell lymphoma, *BCL5*, encodes a Krüppel like zinc-finger protein Blood 1994 1 26 32 8274740

[B9] Cattoretti G Chang CC Cechova K Zhang J Ye BH BCL-6 protein is expressed in germinal-center B cells Blood 1995 1 45 53 7795255

[B10] Yamochi T Kitabayashi A Hirokawa M Miura AB Onizuka T Mori S Regulation of BCL-6 gene expression in human myeloid/monocytoid leukemic cells Leukemia 1997 1 694 700 10.1038/sj.leu.2400631 9180294

[B11] Baron BW Stanger RR Hume E Sadhu A Mick R Kerckaert JP *BCL6 *encodes a sequence-specific DNA-binding protein Genes Chromosomes Cancer 1995 1 221 224 10.1002/gcc.2870130314 7669744

[B12] Seyfert VL Allman D He Y Staudt LM Transcriptional repression by the proto-oncogene BCL-6 Oncogene 1996 1 2331 2342 8649773

[B13] Gupta S Jiang M Anthony A Pernis AB Lineage-specific modulation of interleukin 4 signaling by interferon regulatory factor 4 J Exp Med 1999 1 1837 1848 10.1084/jem.190.12.1837 10601358 PMC2195723

[B14] Harris MB Chang CC Berton MT Danial NN Zhang J Kuehner D Transcriptional repression of Stat6-dependent interleukin-4-induced genes by BCL-6: specific regulation of iepsilon transcription and immunoglobulin E switching Mol Cell Biol 1999 1 7264 7275 10490661 10.1128/mcb.19.10.7264 PMC84719

[B15] Arima M Toyama H Ichii H Kojima S Okada S Hatano M A putative silencer element in the *IL-5 *gene recognized by Bcl6 J Immunol 2002 1 829 836 12097386 10.4049/jimmunol.169.2.829

[B16] Kusam S Toney LM Sato H Dent AL Inhibition of Th2 differentiation and GATA-3 expression by BCL-6 J Immunol 2003 1 2435 2441 12594267 10.4049/jimmunol.170.5.2435

[B17] Takeda N Arima M Tsuruoka N Okada S Hatano M Sakamoto A Bcl6 is a transcriptional repressor for the IL-18 gene J Immunol 2003 1 426 431 12817026 10.4049/jimmunol.171.1.426

[B18] Yu RY Wang X Pixley FJ Yu JJ Dent AL Broxmeyer HE BCL-6 negatively regulates macrophage proliferation by suppressing autocrine IL-6 production Blood 2005 1 1777 1784 10.1182/blood-2004-08-3171 15507530

[B19] Toney LM Cattoretti G Graf JA Merghoub T Pandolfi PP Dalla-Favera R BCL-6 regulates chemokine gene transcription in macrophages Nat Immunol 2000 1 214 220 10.1038/79749 10973278

[B20] Shaffer AL Yu X He Y Boldrick J Chan EP Staudt LM BCL-6 represses genes that function in lymphocyte differentiation, inflammation and cell cycle control Immunity 2000 1 199 212 10.1016/S1074-7613(00)00020-0 10981963

[B21] Niu H Cattoretti G Dalla-Favera R BCL6 controls the expression of the B7-1/CD80 costimulatory receptor in germinal center B cells J Exp Med 2003 1 211 221 10.1084/jem.20021395 12860928 PMC2194068

[B22] Phan RT Saito M Basso K Niu H Dalla-Favera R BCL6 interacts with the transcription factor Miz-1 to suppress the cyclin-dependent kinase inhibitor p21 and cell cycle arrest in germinal center B cells Nat Immunol 2005 1 1054 1060 10.1038/ni1245 16142238

[B23] Phan RT Dalla-Favera R The BCL6 proto-oncogene suppresses p53 expression in germinal-centre B cells Nature 2004 1 635 659 10.1038/nature03147 15577913

[B24] Shvarts A Brummelkamp TR Scheeren F Koh E Daley GQ Spits H A senescence rescue screen identifies BCL6 as an inhibitor of anti-proliferative p19(ARF)-p53 signaling Genes Dev 2002 1 681 686 10.1101/gad.929302 11914273 PMC155362

[B25] Ranuncolo SM Polo JM Dierov J Singer M Kuo T Greally J Bcl-6 mediates the germinal center B cell phenotype and lymphomagenesis through transcriptional repression of the DNA-damage sensor ATR Nat Immunol 2007 1 705 714 10.1038/ni1478 17558410

[B26] Baron BW Zeleznik-Le N Baron MJ Theisler C Huo D Krasowski MD Repression of the *PDCD2 *gene by BCL6 and the implications for the pathogenesis of human B and T cell lymphomas Proc Natl Acad Sci USA 2007 1 7449 7454 10.1073/pnas.0701770104 17468402 PMC1863460

[B27] Baron BW Anastasi J Thirman MJ Furukawa Y Fears S Kim DC The human programmed cell death-2 (*PDCD2*) gene is a target of BCL6 repression: implications for a role of BCL6 in the down-regulation of apoptosis Proc Natl Acad Sci USA 2002 1 2860 2865 10.1073/pnas.042702599 11854457 PMC122438

[B28] Tang TT Dowbenko D Jackson A Toney L Lewin DA Dent AL The forkhead transcription factor AFX activates apoptosis by induction of the BCL-6 transcriptional repressor J Biol Chem 2002 1 14255 14265 10.1074/jbc.M110901200 11777915

[B29] Li Z Wang X Yu RY Ding BB Yu JJ Dai XM BCL-6 negatively regulates expression of the NF-kappaB1 p105/p50 subunit J Immunol 2005 1 205 214 15611242 10.4049/jimmunol.174.1.205

[B30] Harris MB Mostecki J Rothman PB Repression of an interleukin-4-responsive promoter requires cooperative BCL-6 function Biol Chem 2005 1 13114 13121 10.1074/jbc.M412649200 15659391

[B31] Harris MB Chang CC Berton MT Danial NN Zhang J Kuehner D Transcriptional repression of Stat6-dependent interleukin-4-induced genes by BCL-6: specific regulation of iepsilon transcription and immunoglobulin E switching Mol Cell Biol 1999 1 7264 7275 10490661 10.1128/mcb.19.10.7264 PMC84719

[B32] Albagli O Lantoine D Quief S Quignon F Englert C Kerckaert JP Overexpressed BCL6 (LAZ3) oncoprotein triggers apoptosis, delays S phase progression and associates with replication foci Oncogene 1999 1 5063 5075 10.1038/sj.onc.1202892 10490843

[B33] Yamochi T Kaneita Y Akiyama T Mori S Moriyama M Adenovirus-mediated high expression of BCL-6 in CV-1 cells induces apoptotic cell death accompanied by down-regulation of BCL-2 and BCL-X(L) Oncogene 1999 1 487 494 10.1038/sj.onc.1202334 9927205

[B34] Toyama H Okada S Hatano M Takahashi Y Takeda N Ichii H Memory B cells without somatic hypermutation are generated from Bcl6-deficient B cells Immunity 2002 1 329 339 10.1016/S1074-7613(02)00387-4 12354385

[B35] Ichii H Sakamoto A Hatano M Okada S Toyama H Taki S Role for Bcl-6 in the generation and maintenance of memory CD8+ T cells Nat Immunol 2002 1 558 563 10.1038/ni802 12021781

[B36] Ichii H Sakamoto A Kuroda Y Tokuhisa T Bcl6 acts as an amplifier for the generation and proliferative capacity of central memory CD8+ T Cells J Immunol 2004 1 883 891 15240675 10.4049/jimmunol.173.2.883

[B37] Ichii H Sakamoto A Arima M Hatano M Kuroda Y Tokuhisa T Bcl6 is essential for the generation of long-term memory CD4+ T cells Int Immunol 2007 1 427 433 10.1093/intimm/dxm007 17307796

[B38] Allman D Jain A Dent A Maile RR Selvaggi T Kehry MR BCL-6 expression during B-cell activation Blood 1996 1 5257 5268 8652841

[B39] Fero ML Rivkin M Tasch M Porter P Carow CE Firpo E A syndrome of multiorgan hyperplasia with features of gigantism, tumorigenesis, and female sterility in p27(Kip1)-deficient mice Cell 1996 1 733 744 10.1016/S0092-8674(00)81239-8 8646781

[B40] Fero ML Randel E Gurley KE Roberts JM Kemp CJ The murine gene p27Kip1 is haplo-insufficient for tumour suppression Nature 1998 1 177 180 10.1038/24179 9823898 PMC5395202

[B41] Arguni E Arima M Tsuruoka N Sakamoto A Hatano M Tokuhisa T JunD/AP-1 and STAT3 are the major enhancer molecules for high Bcl6 expression in germinal center B cells Int Immunol 2006 1 1079 1089 10.1093/intimm/dxl041 16702165

[B42] Kitayama D Sakamoto A Arima M Hatano M Miyazaki M Tokuhisa T A role for Bcl6 in sequential class switch recombination to IgE in B cells stimulated with IL-4 and IL-21 Mol Immunol 2008 1 1337 1345 10.1016/j.molimm.2007.09.007 17950876

[B43] Yoshida T Fukuda T Hatano M Koseki H Okabe S Ishibashi K A role of Bcl6 in mature cardiac myocytes Cardiovasc Res 1999 1 670 679 10.1016/S0008-6363(99)00007-3 10533607

[B44] Lund R Ahlfors H Kainonen E Lahesmaa AM Dixon C Lahesmaa R Identification of genes involved in the initiation of human Th1 or Th2 cell commitment Eur J Immunol 2005 1 3307 3319 10.1002/eji.200526079 16220538

[B45] Lund RJ Ylikoski EK Aittokallio T Nevalainen O Lahesmaa R Kinetics and STAT4- or STAT6-mediated regulation of genes involved in lymphocyte polarization to Th1 and Th2 cells Eur J Immunol 2003 1 1105 1116 10.1002/eji.200323899 12672077

[B46] Zhou G Ono SJ Induction of BCL-6 gene expression by interferon-gamma and identification of an IRE in exon I Exp Mol Pathol 2005 1 25 35 10.1016/j.yexmp.2004.08.008 15596057

[B47] Kaplan MH Schindler U Smiley ST Grusby MJ Stat6 is required for mediating responses to IL-4 and for development of Th2 cells Immunity 1996 1 313 319 10.1016/S1074-7613(00)80439-2 8624821

[B48] Dent AL Hu-Li J Paul WE Staudt LM T helper type 2 inflammatory disease in the absence of interleukin 4 and transcription factor STAT6 Proc Natl Acad Sci USA 1998 1 13823 13828 10.1073/pnas.95.23.13823 9811885 PMC24910

[B49] Yamaguchi Y Hayashi Y Sugama Y Miura Y Kasahara T Kitamura S Highly purified murine interleukin 5 (IL-5) stimulates eosinophil function and prolongs in vitro survival. IL-5 as an eosinophil chemotactic factor J Exp Med 1988 1 1737 1742 10.1084/jem.167.5.1737 2835420 PMC2188945

[B50] Karpus WJ Lukacs NW Kennedy KJ Smith WS Hurst SD Barrett TA Differential CC chemokine-induced enhancement of T helper cell cytokine production J Immunol 1997 1 4129 4136 9126972

[B51] Gu L Tseng S Horner RM Tam C Loda M Rollins BJ Control of TH2 polarization by the chemokine monocyte chemoattractant protein-1 Nature 2000 1 407 411 10.1038/35006097 10746730

[B52] Braun MC Lahey E Kelsall BL Selective suppression of IL-12 production by chemoattractants J Immunol 2000 1 3009 3017 10706689 10.4049/jimmunol.164.6.3009

[B53] Okamura H Tsutsi H Komatsu T Yutsudo M Hakura A Tanimoto T Cloning of a new cytokine that induces IFN-gamma production by T cells Nature 1995 1 88 91 10.1038/378088a0 7477296

[B54] Nakanishi K Yoshimoto T Tsutsi H Okamura H Interleukin-18 is a unique cytokine that stimulates both Th1 and Th2 responses depending on its cytokine milieu Cytokine Growth Factor Rev 2001 1 53 72 10.1016/S1359-6101(00)00015-0 11312119

[B55] Sugimoto T Ishikawa Y Yoshimoto T Hayashi N Fujimoto J Nakanishi K Interleukin 18 acts on memory T helper cells type 1 to induce airway inflammation and hyperresponsiveness in a naive host mouse J Exp Med 2004 1 535 545 10.1084/jem.20031368 14970180 PMC2211833

[B56] El-Mezzein RE Matsumoto T Nomiyama H Miike T Increased secretion of IL-18 in vitro by peripheral blood mononuclear cells of patients with bronchial asthma and atopic dermatitis J Clin Immunol 2001 1 193 198 10.1046/j.1365-2249.2001.01664.x PMC1906188 11703360

[B57] Yoshizawa Y Nomaguchi H Izaki S Kitamura K Serum cytokine levels in atopic dermatitis Clin Exp Dermatol 2002 1 225 229 10.1046/j.1365-2230.2002.00987.x 12072014

[B58] Wong CK Ho CY Ko FW Chan CH Ho AS Hui DS Proinflammatory cytokines (IL-17, IL-6, IL-18 and IL-12) and Th cytokines (IFN-g, IL-4, IL-10 and IL-13) in patients with allergic asthma Clin Exp Immunol 2001 1 177 183 10.1046/j.1365-2249.2001.01602.x 11529906 PMC1906135

[B59] Tanaka H Miyazaki N Oashi K Teramoto S Shiratori M Hashimoto M IL-18 might reflect disease activity in mild and moderate asthma exacerbation J Allergy Clin Immunol 2001 1 331 336 10.1067/mai.2001.112275 11174201

[B60] Higashi N Gesser B Kawana S Thestrup-Pedersen K Expression of IL-18 mRNA and secretion of IL-18 are reduced in monocytes from patients with atopic dermatitis J Allergy Clin Immunol 2001 1 607 614 10.1067/mai.2001.118601 11590389

[B61] Verhaeghe B Gevaert P Holtappels G Lukat KF Lange B Van Cauwenberge P Up-regulation of IL-18 in allergic rhinitis Allergy 2002 1 825 830 10.1034/j.1398-9995.2002.23539.x 12169180

[B62] Levi-Schaffer F Temkin V Malamud V Feld S Zilberman Y Mast cells enhance eosinophil survival in vitro: role of TNF-alpha and granulocytemacrophage colony-stimulating factor J Immunol 1998 1 5554 5562 9605160

[B63] Skokos S Le Panse S Villa I Rousselle JC Peronet R David B Mast cell-dependent B and T lymphocyte activation is mediated by the secretion of immunologically active exosomes J Immunol 2001 1 868 876 11145662 10.4049/jimmunol.166.2.868

[B64] Skokos D Botros HG Demeure C Morin J Peronet R Birkenmeier G Mast cell-derived exosomes induce phenotypic and functional maturation of dendritic cells and elicit specific immune responses in vivo J Immunol 2003 1 3037 3045 12626558 10.4049/jimmunol.170.6.3037

[B65] Schmitz J Thiel A Kuhn R Rajewsky K Muller W Assenmacher M Induction of interleukin 4 (IL-4) expression in T helper (Th) cells is not dependent on IL-4 from non-Th cells J Exp Med 1994 1 1349 1353 10.1084/jem.179.4.1349 8145047 PMC2191446

[B66] Fallon PG Jolin HE Smith P Emson CL Townsend MJ Fallon R IL-4 induces characteristic Th2 responses even in the combined absence of IL-5, IL-9, and IL-13 Immunity 2002 1 7 17 10.1016/S1074-7613(02)00332-1 12150887

[B67] Nakajima T Inagaki N Tanaka H Tanaka A Yoshikawa M Tamari M Marked increase in CC chemokine gene expression in both human and mouse mast cell transcriptomes following Fcepsilon receptor I cross-linking: an interspecies comparison Blood 2002 1 3861 3868 10.1182/blood-2002-02-0602 12393595

[B68] McKenzie GJ Emson CL Bell SE Anderson S Fallon P Zurawski G Impaired development of Th2 cells in IL-13-deficient mice Immunity 1998 1 423 432 10.1016/S1074-7613(00)80625-1 9768762

[B69] Chiaramonte G Donaldson DD Cheever AW Wynn TA An IL-13 inhibitor blocks the development of hepatic fibrosis during a T-helper type 2-dominated inflammatory response J Clin Invest 1999 1 777 785 10.1172/JCI7325 10491413 PMC408441

[B70] Adra CN Gao PS Mao XQ Baron BW Pauker S Miki T Variants of B cell lymphoma 6 (BCL6) and marked atopy Clin Genet 1998 1 362 364 9831352 10.1034/j.1399-0004.1998.5440418.x

[B71] O'Garra A Cytokines induce the development of functionally heterogeneous T helper cell subsets Immunity 1998 1 275 283 10.1016/S1074-7613(00)80533-6 9529145

